# Bta-Let-7d Modulation of Oxidative Stress Induced by Potassium Permanganate in Bovine Endometrial Cells via IGF1R/PI3K/AKT Signaling Pathway

**DOI:** 10.3390/antiox14040444

**Published:** 2025-04-08

**Authors:** Wenjing Liu, Talha Umar, Wen Feng, Bohan Zhang, Jinxin Zhang, Han Zhou, Nuoer Chen, Ganzhen Deng, Siyu Xiao

**Affiliations:** Department of Clinical Veterinary Medicine, College of Veterinary Medicine, Huazhong Agricultural University, Wuhan 430070, China; lwj0916@webmail.hzau.edu.cn (W.L.); talhaumar@webmail.hzau.edu.cn (T.U.); fengwen@webmail.hzau.edu.cn (W.F.); 2022302110218@webmail.hzau.edu.cn (B.Z.); zhangjinxin@webmail.hzau.edu.cn (J.Z.); zhouhan@webmail.hzau.edu.cn (H.Z.); cne@webmail.hzau.edu.cn (N.C.)

**Keywords:** potassium permanganate, bta-let-7d, IGF1R, oxidative stress, dairy cows

## Abstract

Oxidative stress is a significant factor affecting reproductive efficiency in dairy cows, contributing to conditions such as endometritis that impair fertility and milk production. This study investigates the molecular mechanisms by which bta-let-7d modulates the oxidative stress responses induced by potassium permanganate (KMnO_4_) in bovine endometrial epithelial cells (BEECs). Using KMnO_4_ to induce oxidative stress, we observed significant increases in reactive oxygen species (ROS) and malondialdehyde (MDA) levels, accompanied by decreased activities of the antioxidant enzymes glutathione peroxidase (GPx) and superoxide dismutase (SOD). Quantitative PCR and Western blot analyses indicated a negative correlation between IGF1R and bta-let-7d expression in oxidative-stress-affected tissues, suggesting opposing roles in managing stress responses. Following KMnO_4_ treatment, there was marked downregulation of anti-apoptotic genes and an upregulation of pro-apoptotic markers, alongside diminished antioxidant capacity. Mechanistically, bta-let-7d targets IGF1R, leading to the suppression of the PI3K/AKT signaling pathway and exacerbating oxidative damage. In vivo experiments further confirmed the impact of KMnO_4_ exposure on IGF1R expression. These findings provide novel insights into the mechanisms by which KMnO_4_ induces oxidative stress and apoptosis in bovine uterus. They highlight the potential for therapeutic strategies targeting the bta-let-7d/IGF1R axis to enhance reproductive health management in dairy cows, offering a promising avenue for mitigating oxidative-stress-related reproductive disorders.

## 1. Introduction

Oxidative stress plays a crucial role in the pathophysiology of various reproductive disorders in dairy cows, particularly in cases of postpartum uterine infections, such as endometritis [1]. These infections are primarily caused by bacterial contaminants such as *Escherichia coli* and *Staphylococcus aureus*, which disrupt the normal function of the endometrium and create an unsuitable environment for embryo implantation, ultimately reducing reproductive performance [2,3,4]. The accumulation of reactive oxygen species (ROS) can lead to cellular damage and impaired lipid peroxidation health; therefore, studying the effects of oxidative stress on the health of the dairy cow endometrium is meaningful [5].

Acute endometritis in dairy cows may be treated with potassium permanganate (KMnO_4_), recognized for its antibacterial and anti-inflammatory properties [6]. However, KMnO_4_ is a strong oxidizing agent, and incomplete uterine washing can result in residual potassium permanganate within the body, potentially creating a prolonged oxidative environment that may lead to oxidative damage of the endometrium [7,8,9,10]. In the context of postpartum infections in dairy cows, KMnO_4_ is utilized to cleanse the uterus, disinfect wounds, and treat hoof conditions [11,12]. Additionally, it is employed in other animal species for wound debridement and the management of uterine adenomyosis in canines and felines. Nonetheless, potassium permanganate’s potent oxidative properties raise concerns, as it has been shown to exhibit acute toxicity in rodent models [13,14,15]. This study aims to investigate the potential for potassium permanganate to induce oxidative stress in the endometrium during therapeutic application.

IGF1R (Insulin-like Growth Factor 1 Receptor) is essential in oxidative stress [16]. It belongs to the receptor tyrosine kinase family. It can be activated by IGF1 or IGF2 [17], which activate its downstream PI3K/AKT (Phosphoinositide 3-Kinase/Protein Kinase B) signaling pathway, regulating cell growth, differentiation, and responses to oxidative stress [18]. IGF1R is significantly related to oxidative stress responses, particularly in the context of ischemia/reperfusion injury and mental disorders. Studies have shown that low concentrations of H_2_O_2_ during myocardial ischemia–reperfusion activate the IGF1R/PI3K/AKT signaling pathway to resist oxidative stress damage and prevent apoptosis [19,20,21].

MicroRNAs (miRNAs) are a class of small non-coding RNAs that regulate gene expression by binding to the 3′ untranslated regions of mRNAs, leading to mRNA degradation or the inhibition of translation [22]. The let-7 family is a group of conserved microRNAs that play roles in various biological processes, including cell proliferation, differentiation, and apoptosis. The let-7 family is highly expressed in mitochondria and acts by regulating antioxidant-related genes [23]. For example, let-7b and let-7c can indirectly regulate HMOX1 (Heme Oxygenase 1) to alleviate oxidative damage in human hepatocytes [24]. Studies have shown that let-7 family member let-7a inhibits oxidative stress and cellular damage by targeting arginase 2 (ARG2) [25]. The let-7 family also plays a significant role in regulating cancer stem cells, participating in the maintenance of stem cell properties, promoting the proliferation of cancer cells, and resisting apoptosis [26,27]. Let-7d was selected due to its high expression in endometrial tissue and its significant role in mitochondrial function [28,29].

This research aims to investigate the implications of potassium permanganate treatment on oxidative stress in the endometrium of dairy cows. By examining the interactions between oxidative stress, IGF1R signaling, and miRNA regulation in the context of reproductive health, this study seeks to provide insights into potential therapeutic strategies for managing oxidative-stress-related reproductive disorders in dairy cows. Understanding these mechanisms may pave the way for improved reproductive outcomes and economic viability in the dairy industry.

## 2. Material and Methods

### 2.1. Collection of Bovine Uterine Samples and SD Rat Model

Bovine uterine tissue samples were obtained from a slaughterhouse in Wuhan, China. Following collection, after being cut with a scalpel into pieces, the tissues were either immediately immersed in liquid nitrogen or kept in pre-cooled PBS containing 5% antibiotics, and transported to the laboratory within 30 min. Cows with clinical signs of purulent vaginal discharge and visibly pathological changes in the uterus during necropsy were selected for the endometritis group. A total of 6 samples of uterine tissue were collected from endometritis cows and 6 samples from the healthy cow group. Tissues were either fixed in 50 mL of 4% paraformaldehyde for subsequent H&E staining, immunohistochemistry, and immunofluorescence analysis, or immersed in PBS-based sample preservation solution for cell isolation.

All animal procedures adhered to protocols approved by the Hubei Province Laboratory Animal Research Center and the Animal Research Ethics Committee of Huazhong Agricultural University (HZAUMO-2015-12). Additionally, 4-to-6-week-old Sprague Dawley (SD) female rats were procured from the Experimental Animal Center. The rats were housed under standard conditions with a 12 h light/dark cycle, fed a standard diet, and provided with water ad libitum for an acclimatization period of 7 days. The SD rats were randomly divided into 4 groups (*n* = 5 per group). The euthanasia procedure was performed in accordance with the guidelines for the humane euthanasia of laboratory animals. For adult rats, intraperitoneal injection of phenobarbital sodium at a dose of 40 mg/kg was used for anesthesia. Briefly, the rats were placed in the supine position on the operating table after anesthesia. The abdominal area was shaved and cleaned with 70% ethanol. A midline abdominal incision was made to expose the uterine horns. The uterine horns were flushed with varying concentrations of KMnO_4_ using a sterile syringe.

Both uterine horns of the rats were exposed and flushed with varying concentrations of potassium permanganate (KMnO_4_). The specific concentrations used were 0.01%, 0.025% and 0.05% KMnO_4_, designated as C1, C2, and C3, respectively, while the control group received an equivalent volume of phosphate-buffered saline (PBS) via injection with a total volume of 20 µL. Each concentration was administered for a duration of 5 min, followed by gentle rinsing with sterile saline to remove residual KMnO_4_. After flushing, small tissue samples were excised from the uterine horns for histological analysis. At the end of the experiment, the rats were euthanized using carbon dioxide inhalation followed by cervical dislocation to ensure death.

### 2.2. Histological Analysis

The uterine samples from both the bovines and SD rats were fixed in 10% formalin, followed by washing with a buffer (e.g., PBS), dehydration through a graded ethanol series, clearing with xylene, and embedding in molten paraffin. The embedded tissues were placed in molds and allowed to solidify at room temperature or in cold storage. The solidified blocks were sectioned into thin slices (4–6 μm) using a microtome, deparaffinized with xylene, and stained with hematoxylin for nuclei and eosin for cytoplasmic visualization.

### 2.3. Bovine Endometrial Epithelial Cell Culture

Bovine endometrial epithelial cells (BEECs) were isolated from the uterine cornua tissues of cows around 30 months old. Briefly, take 0.5–2 g of bovine uterine horn segment (1–2 cm in length) and rinse it three times in DPBS (Dulbecco’s Phosphate-Buffered Saline). Longitudinally dissect the uterine horn to expose the endometrium. Immerse the tissue in DPBS containing 5% penicillin/streptomycin and incubate it for 30–60 min to reduce contamination. Submerge the dissected tissue completely in a 1% trypsin solution and digest it at 4 °C for 16–20 h. This prolonged digestion at low temperature ensures effective cell isolation while minimizing tissue damage. After digestion, rinse the tissue three times with DPBS to remove residual trypsin. Mechanically scrape the endometrial epithelial cells from the tissue surface. Centrifuge the collected cells at 1200 r/min for 5 min. Repeat the centrifugation process three times to ensure purity. Resuspend the isolated cells in DMEM/F12 culture medium supplemented with 15% FBS (fetal bovine serum) and 1% penicillin/streptomycin. Adjust the cell concentration to 1 × 10^5^ cells/mL. Plate the cells and culture them at 37 °C in a humidified incubator with 5% CO_2_. Change the medium every 1–2 days. BEECs can typically be obtained and used for experiments within 3–5 days of culture.

HEK293T cells (human embryonic kidney cell line) were purchased from the American Type Culture Collection (ATCC CRL-2398™) and were cultured in a medium supplemented with 10% fetal bovine serum (Sigma, St. Louis, MO, USA) and 1% penicillin/streptomycin (Biosharp, Beijing, China). The incubation environment was maintained at 5% CO_2_ and 37 °C, with medium changes every 6 h. Upon reaching approximately 80% confluence, the cells were digested with trypsin, subcultured, and stored at −80 °C or in liquid nitrogen. To investigate the role of let-7d and IGF1R in oxidative stress within the bovine endometrium, an in vitro oxidative stress model was established by treating the cells with 0.01% KMnO_4_.

### 2.4. BEEC Transfection

When cell cultures reached approximately 60% confluence, transfections were performed using the jetPRIME^®^ reagent (Polyplus, Strasbourg, France). Briefly, after washing three times with sterile PBS, cells of each well were replaced with 1.5 mL of OptiMEM medium (Thermo Fisher Scientific, Waltham, MA, USA) and returned to the incubator for further incubation. The lipid mixture was prepared with an aliquot of 500 ng of recombinant pmirGLO plasmid, and 5 μL of let-7d mimic (final concentration 20 μM) was added to 100 μL of jetPRIME^®^ buffer. After vigorous shaking and inversion, the mixture was centrifuged and 4 μL of jetPRIME was added. Following another 10s of vigorous shaking and centrifugation, the mixture was left at room temperature for 10 min. The lipid mixture was added to the cells at 200 μL per well, and the cell culture plate was gently shaken to ensure even distribution. The cell plate was incubated at 37 °C with 5% CO_2_ for 6 h, after which the medium was replaced with fresh normal culture medium and the cells were incubated for another 24–48 h before subsequent experiments. bta-let-7d mimics (5′–3′: S: AGAGGUAGUAGGUUGCAUAGUU, AS: CUAUGCAACCUACUACCUCUUU), bta-let-7d inhibitor (5′–3′: S: AACUAUGCAACCUACUACCUCU), and IGF1R siRNA (si-IGF1R, 5′–3′: S: GCACAACUACUGCUCCAAATT, AS: UUUGGAGCAGUAGUUGUGCTT) were used. RNA and protein were extracted 24 h post-transfection.

### 2.5. RNA Extraction and RT-qPCR Analysis

Total RNA from tissues or cells was extracted using Trizol reagent according to the manufacturer’s instructions. Complementary DNA (cDNA) synthesis was conducted using the Hifair^®^ AdvanceFast One-step RT-gDNA Digestion Super Mix kit (Yeasen, Shanghai, China) following the protocol, with miRNA reverse transcription achieved through tailing (SYBR Green Master Mix, Yeasen, Shanghai, China). Gene expression was quantified via real-time PCR (LC96, Roche, Shanghai, China). β-actin was used as the internal control for mRNA and U6 was used as the internal control for miRNAs. The relative gene expression levels were analyzed using the 2^−ΔΔCT^ method. Primer sequences are provided in Table 1.

### 2.6. Western Blot Analysis

Following protein extraction from tissues and cells using RIPA buffer (1%Triton X-100, 1% sodium deoxycholate, 0.1% SDS) with phosphatase inhibitors (PMSF, at a ratio of 100:1:1) (Lot: ST505, Beyotime, Beijing, China), the protein concentrations in each group were quantified using a BCA assay (HYCEZMBIO, Wuhan, China). Samples underwent SDS-PAGE under constant voltage at 100 v with a current of 20–30 mA for 1.5 h, followed by transfer to PVDF membranes (Millipore, Billerica, MA, USA) using constant current. Membranes were blocked with ECL (Affinity, Cincinnati, OH, USA) rapid blocking buffer for one hour and incubated overnight with primary antibodies at 4 °C (16–18 h). The primary antibodies were anti-AKT (Abmart, Shanghai, China, Lot: T55561); anti-p-AKT (Abmart, Shanghai, China, Lot:T40067); anti-PI3K (Abmart, Shanghai, China, Lot: T40064); anti-p-PI3K (Abmart, Shanghai, China, Lot: T40116); anti-Bax (Abmart, Shanghai, China, Lot: T40051); anti-Bcl-2 (Proteintech, Wuhan, China, Lot: 68103-1-lg); anti β-actin (Abclonal, Wuhan, China, Lot: AC006). After three washes with TBST, membranes were incubated with secondary antibodies (HRP Goat Anti-Rabbit IgG, Lot: AS014; HRP Goat Anti-Mouse IgG Lot: AS003; Abclonal, Wuhan, China) for two hours, rewashed thrice with TBST, and visualized (Fujifilm, Tokyo, Japan). The results were analyzed using ImageJ2 software. p-PI3K uses PI3K as an internal control and P-AKT uses AKT as an internal control; other proteins use β-actin as an internal control.

### 2.7. miRNA Target Prediction

To explore the molecular action of IGF1R, the potential miRNA targets of IGF1R were predicted using the TargetScan website, and let-7d was selected for further study. These miRNAs were further assessed based on their expression in bovine endometrial tissue and previous studies regarding their functions. Additional target predictions were conducted using miRDB (http://mirdb.org/, accessed on 10 October 2023), PicTar (https://pictar.mdc-berlin.de/, accessed on 10 October 2023), and miRcode (http://www.mircode.org/, accessed on 10 October 2023). Target prediction (TargetScan, miRDB) identified IGF1R as a putative bta-let-7d target, guiding validation in luciferase assays.

### 2.8. Dual-Luciferase Reporter Assay

Wild-type and mutant plasmids for IGF1R were constructed using the primGLO backbone based on predicted binding sites. HEK293T cells were seeded into a 6-well plate and transfection was initiated when the cell density reached 80%. The cell transfection procedure was performed according to the BEEC transfection protocol in Section 2.4. These plasmids, along with bta-let-7d mimics, were co-transfected into 293T cells. Fluorescence intensity was measured using the Dual-Luciferase Assay Kit (Cat. No. 11402ES60, Yeasen, China).

### 2.9. Detection of MDA, ROS, T-AOC, GPx, and SOD

After stimulation with KMnO_4_ (C1: 0.01%, C2: 0.025%, C3: 0.05%, C4: 0.1%, C5:0.5%) for 12 h, BEECs from each group were washed with PBS and collected using a scraper in 500 μL PBS. Supernatants were obtained following centrifugation for 20 min, and protein concentrations were determined via BCA assay (Lot: HBCA-500, HYCEZMBIO, China). Protein concentration was determined by measuring the absorbance at 562 nm. Standard and sample solutions were prepared, and the absorbance was measured using a spectrophotometer-PE Enspire (PerkinElmer, Waltham, MA, USA). A standard curve was plotted with the concentration of standard solutions on the x-axis and the corresponding absorbance on the y-axis. The concentration of the protein sample was calculated using the equation of the standard curve. Assays for MDA (Cat. No. S0131S), ROS (Cat. No. S0033S), total antioxidant capacity (T-AOC) (Cat. No. S0119), glutathione peroxidase (GPx) (Cat. No. S0056) (from Beyotime, Shanghai, China) and total superoxide dismutase (T-SOD) (Cat. No. A001-1-2) (from Nanjing Jiancheng, Nanjing, China) were performed according to the respective kit instructions.

### 2.10. Immunofluorescence Staining

BEECs were seeded onto sterile glass coverslips placed in 24-well plates at a density of 30,000 cells per coverslip. The coverslips were sterilized by autoclaving or by soaking in 70% ethanol and then rinsed with sterile PBS. Following stimulation with KMnO_4_ (C1: 0.01%, C2: 0.025%, C3: 0.05%, C4: 0.1%, C5: 0.5%) for 12 h, cells were washed twice with PBS and fixed with 4% paraformaldehyde for 20 min. Cells were then permeabilized with Triton for 15 min, washed three times, and blocked with blocking buffer at room temperature for three hours. Primary antibodies (anti- IGF1R, 1:200; anti-phosphorylated m-TOR, 1:400) were added and incubated overnight at 4 °C in the dark. After three additional washes with PBS, secondary antibodies (FITC Goat Anti-Rabbit IgG, Lot:AS011, 1:200; or FITC Goat Anti-Mouse IgG, Lot:AS001, 1:200; Abclonal, China) were added and incubated at room temperature for three hours. Nuclei were stained with DAPI at room temperature for 15 min. Cell coverslips were rewashed three times and visualized using a fluorescence microscope (CKX41, Olympus, Tokyo, Japan).

### 2.11. Statistical Analysis

All data were processed using GraphPad 9.4, with each experiment performed in triplicate. Data are presented as mean ± standard error of the mean (SEM). T-tests were employed for comparisons between two groups, while one-way ANOVA was utilized for comparisons among multiple groups. Statistical significance was defined as *p* < 0.05, with highly significant values indicated at *p* < 0.01.

## 3. Result

### 3.1. Differential Expression of IGF1R and Bta-Let-7d in Normal and Oxidative-Stress-Affected Tissues

**Figure 1:**
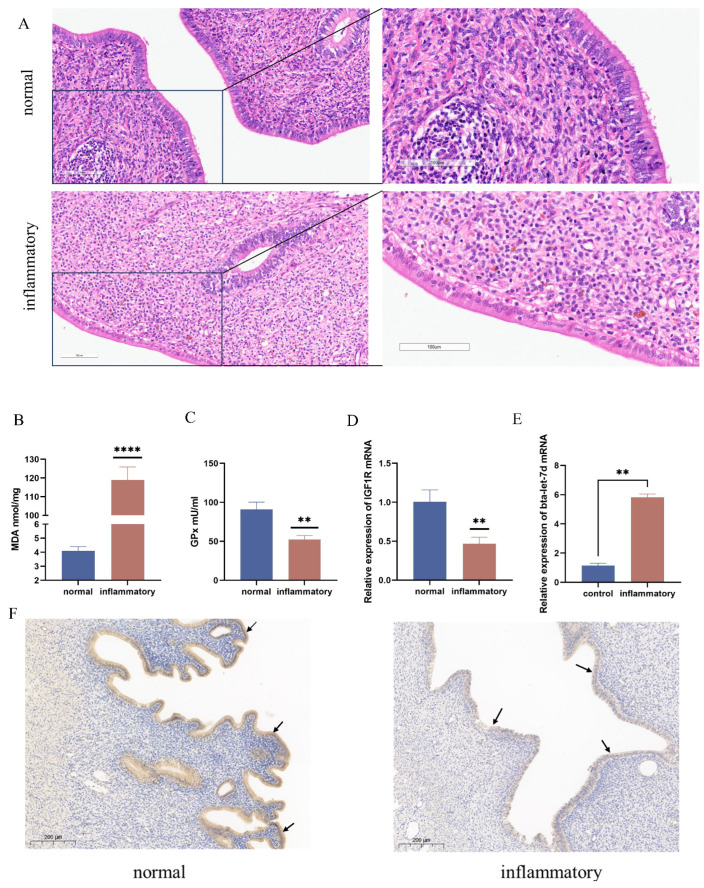
Differential expression of IGF1R and let-7d in normal and oxidative-stress-affected bovine uterine tissues. (**A**) Histopathological analysis of tissues stained with H&E; scale bar = 100 μm. (**B**) Malondialdehyde (MDA) levels in normal and inflammatory tissues were detected. (**C**) Glutathione peroxidase (GPx) levels in normal and inflammatory tissues were detected. (**D**) qPCR was used to measure the expression levels of *IGF1R* mRNA in the tissues. (**E**) qPCR was used to detect the expression levels of bta-let-7d in the tissues. (**F**) Immunohistochemical staining showing nuclear localization (blue) and IGF1R expression in normal and inflammatory bovine uterine tissues. The arrow indicates a reduction in IGF1R protein expression. Data are expressed as the mean ± SEM of three independent experiments. ** *p* < 0.01, **** *p* < 0.0001.

The analysis of bovines’ normal versus oxidative-stress-affected tissues revealed a significant reduction in both mRNA and protein levels of IGF1R in the oxidative stress group, alongside a marked increase in bta-let-7d expression. The histological examination of the oxidative stress group demonstrated considerable cellular damage and necrosis, correlating with the downregulation of IGF1R. In contrast, IGF1R levels were maintained in normal tissues, which exhibited preserved cellular architecture (Figure 1A). Assays measuring malondialdehyde (MDA) and glutathione peroxidase (GPx) levels in normal and inflamed bovine uterine tissues indicated elevated MDA (*p* < 0.001) and reduced GPx levels (*p* < 0.01) in the oxidative stress group compared to normal tissues (Figure 1B,C). Quantitative PCR analysis further confirmed the significant reduction in IGF1R expression (*p* < 0.01) and the notable increase in bta-let-7d expression (*p* < 0.01) in oxidative stress tissues versus normal tissues (Figure 1D,E). Immunohistochemical staining illustrated nuclear localization (blue) and IGF1R expression in both normal and inflamed bovine uterine tissues. The arrow indicates a reduction in IGF1R protein expression (Figure 1F). These findings suggest that oxidative stress markedly alters the expression of IGF1R and bta-let-7d in bovine uterine tissues.

### 3.2. Establishing a KMnO_4_-Induced Oxidative Stress Model in Bovine Endometrial Epithelial Cells

**Figure 2:**
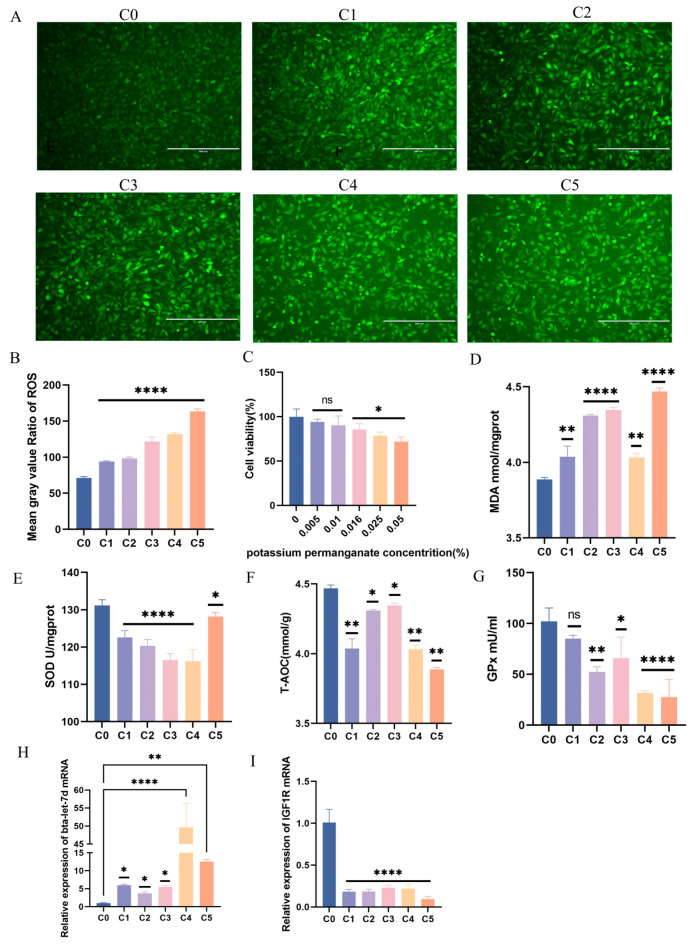
Determination of oxidative stress in BEECs under 12 h effect of 5 different concentrations of potassium permanganate. (**A**,**B**) Fluorescence intensity of ROS in BEECs under different concentrations of potassium permanganate. (**C**) CCK-8 assay detecting cell viability in BEECs exposed to five different concentrations of potassium permanganate for 12 h. (**D**) Lipid peroxidation (MDA) levels in BEECs. (**E**) Total superoxide dismutase (SOD) levels in BEECs. (**F**) Total antioxidant capacity (T-AOC) in BEECs under five concentrations of potassium permanganate. (**G**) Glutathione peroxidase (GPx) levels in BEECs. (**H**,**I**) RT-qPCR was used to detect the relative expression levels of IGF1R and bta-let-7d, with β-actin and U6 as internal controls, respectively. Concentrations for KMnO_4_ are C1: 0.01%, C2: 0.025%, C3: 0.05%, C4: 0.1%, and C5: 0.5%. Data are expressed as the mean ± SEM of three independent experiments. * *p* < 0.05, ** *p* < 0.01, **** *p* < 0.0001.

To assess the oxidative stress induction by KMnO_4_ in BEECs, we analyzed oxidative and antioxidant marker changes across five KMnO_4_ concentrations (C1: 0.01%, C2: 0.025%, C3: 0.05%, C4: 0.1%, C5: 0.5%). Reactive oxygen species (ROS) intensity (Figure 2A,C) exhibited a concentration-dependent increase, indicating a positive correlation between ROS levels and KMnO_4_ concentration (*p* < 0.001). Cell viability, evaluated via the CCK-8 assay (Figure 2B), exhibited a decline in a concentration-dependent manner after 12 h of exposure, reflecting the cytotoxic effects of elevated KMnO_4_ concentrations. Lipid peroxidation, as indicated by MDA levels (*p* < 0.01) (Figure 2D), also significantly increased with rising KMnO_4_ concentrations, further supporting oxidative stress induction. Levels of antioxidant enzymes, including total superoxide dismutase (SOD) (Figure 2E) and total antioxidant capacity (T-AOC) (Figure 2F), decreased significantly following KMnO_4_ exposure, highlighting a compromised antioxidant defense against oxidative damage. Additionally, GPx levels (*p* < 0.001) (Figure 2G) similarly diminished with increasing KMnO_4_ concentrations, indicating reduced cellular antioxidant capacity. Additionally, qPCR results demonstrated that IGF1R and bta-let-7d mRNA expression levels showed opposing trends under varying KMnO_4_ concentrations (Figure 2H,I). These results confirm that KMnO_4_ induces oxidative stress in BEECs in a concentration-dependent manner.

### 3.3. Response Patterns of IGF1R and Bta-Let-7d in the Cell Model

**Figure 3:**
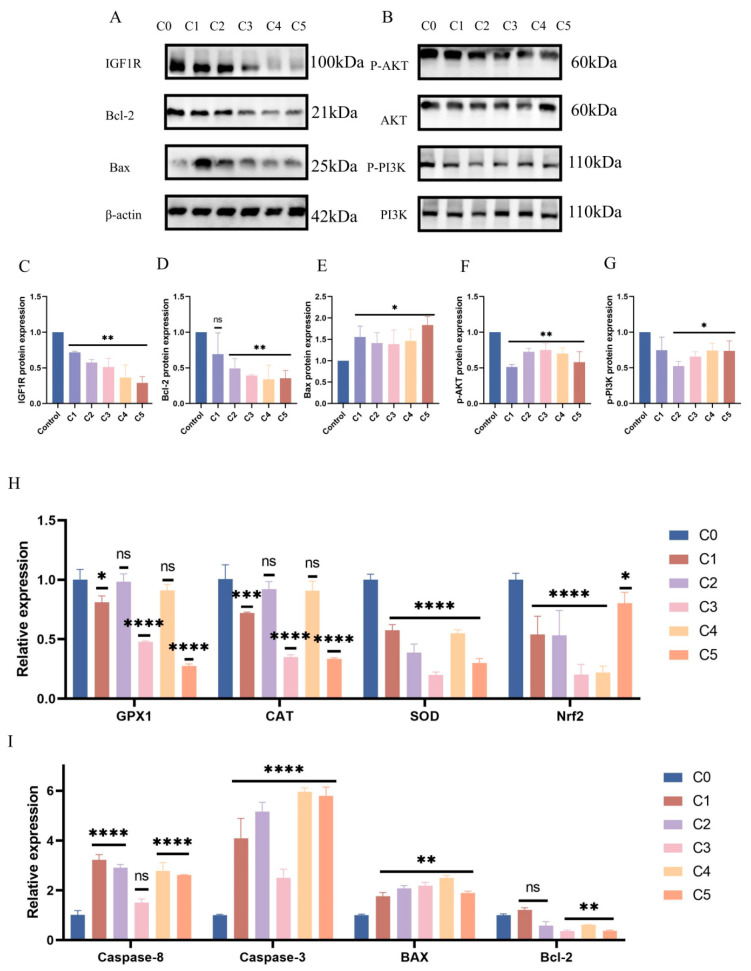

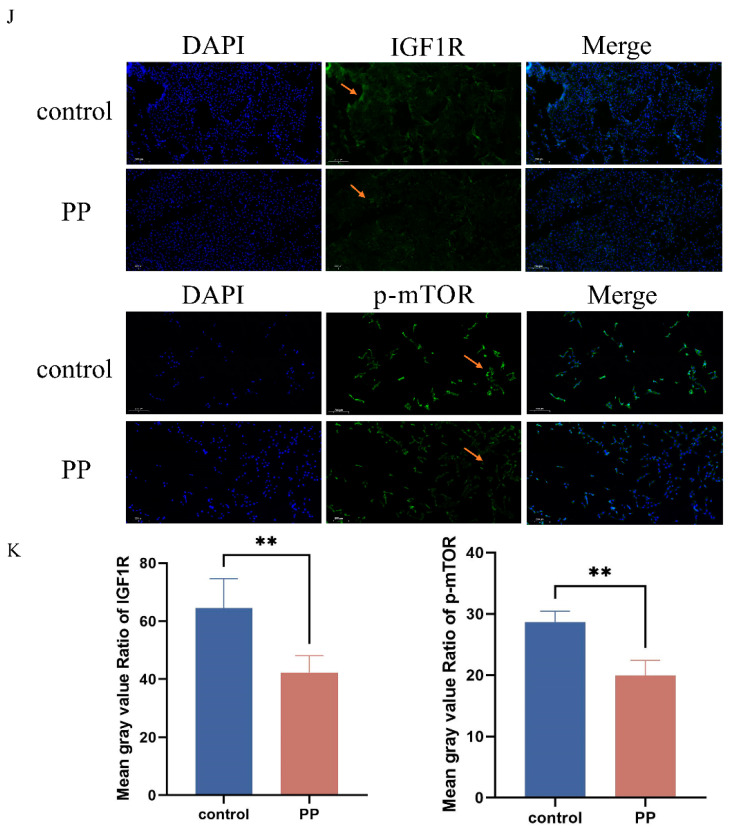
Response patterns of IGF1R and let-7d in BEECs. (**A**–**G**) BEECs were subjected to the same conditions as the previous step. Western blotting was used to detect the protein levels of IGF1R, Bcl-2, Bax, phosphorylated AKT, total AKT, phosphorylated PI3K, and total PI3K, with β-actin as an internal control. The protein bands were quantified using ImageJ software. (**H**,**I**) RT-qPCR was used to measure the relative expression levels of *GPx1*, *CAT*, *SOD*, *Nrf2*, *Caspase-6*, *Caspase-3*, *Bax*, and *Bcl-2*, with β-actin as an internal control. (**J**,**K**) IGF1R and p-mTOR fluorescence intensities were detected in BEECs stimulated by C1. The arrow indicates a reduction in IGF1R and p-mTOR protein expression. Gray values of the indicated protein were detected using Image J gel analysis software. Data are presented as the mean ± SEM of three independent experiments. Concentrations for KMnO_4_ are C1: 0.01%, C2: 0.025%, C3: 0.05%, C4: 0.1%, and C5: 0.5%. PP stands for potassium permanganate (KMnO_4_). * *p* < 0.05; ** *p* < 0.01, *** *p* < 0.001, **** *p* < 0.0001.

Subsequent evaluation of PI3K/AKT signaling pathway protein expression under KMnO_4_ treatment revealed significantly reduced levels of phosphorylated PI3K (*p* < 0.01) and AKT (*p* < 0.05) with increasing KMnO_4_ concentrations (Figure 3A–G), suggesting that oxidative stress may impair cell survival by inhibiting the PI3K/AKT pathway. The expression of apoptosis-related proteins Bcl-2 and Bax was assessed, revealing an increase in Bax (*p* < 0.05) and a decrease in Bcl-2 expression (*p* < 0.01) with higher KMnO_4_ concentrations. Western blot analyses confirmed the decline in IGF1R expression following KMnO_4_ stimulation. Quantitative PCR data indicated significant reductions in GPx1 and CAT expression at KMnO_4_ concentrations of C1, C3, and C5, while SOD and Nrf2 levels were downregulated across all tested KMnO_4_ concentrations. The pro-apoptotic proteins Bax, Caspase-8, and Caspase-3 were upregulated, whereas the expression of the anti-apoptotic protein Bcl-2 decreased. Immunofluorescence results demonstrated diminished IGF1R expression and phosphorylated mTOR levels under KMnO_4_ stimulation. The arrow indicates a reduction in IGF1R and p-mTOR protein expression. These findings suggest that KMnO_4_-induced oxidative stress promotes apoptosis in bovine endometrial epithelial cells through the IGF1R/PI3K/AKT signaling pathway. * *p* < 0.05; ** *p* < 0.01, *** *p* < 0.001, **** *p* < 0.0001.

### 3.4. Regulatory Role of Bta-Let-7d in Oxidative Stress Marker Expression

**Figure 4:**
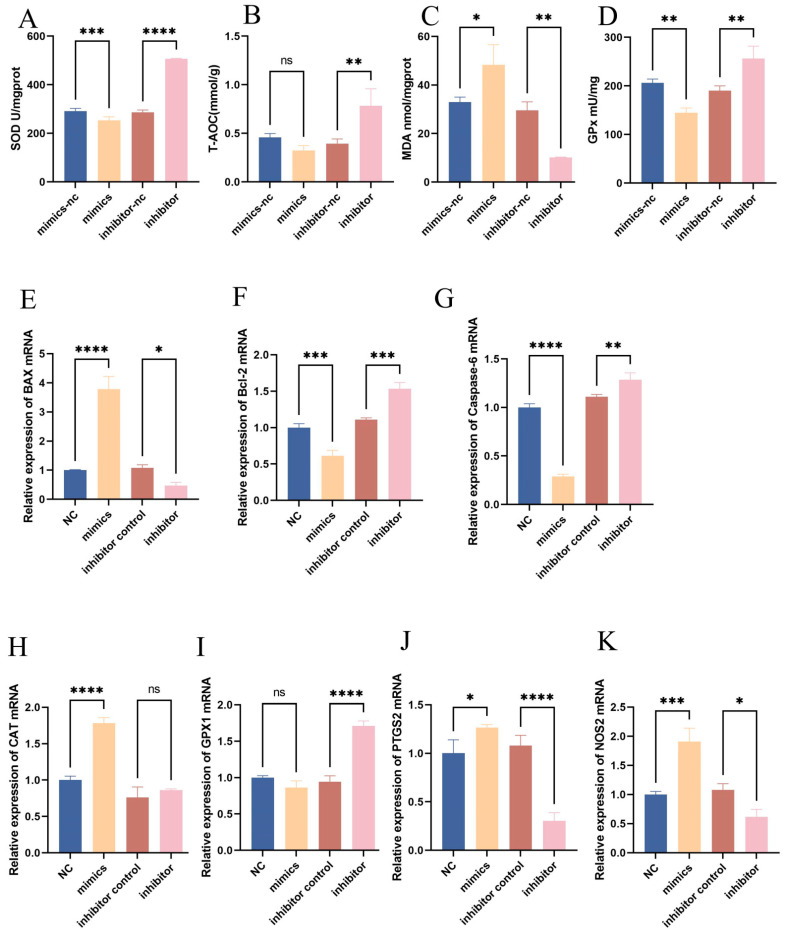
Regulatory role of let-7d in the expression of oxidative stress markers. BEECs were transfected with miRNA mimics and antagonists of bta-let-7d and their respective negative controls. The expression levels of total superoxide dismutase (SOD), total antioxidant capacity (T-AOC), lipid peroxidation (MDA), and glutathione peroxidase (GPx) were measured using commercial kits. (**A**–**D**) RT-qPCR was used to measure the relative expression levels of *Bax*, *Bcl-2*, *Caspase-6*, *CAT*, *GPx1*, *PTGS2*, and *NOS2*, with β-actin as an internal control. (**E**–**K**) Data are presented as the mean ± SEM of three independent experiments. * *p* < 0.05; ** *p* < 0.01, *** *p* < 0.001, **** *p* < 0.0001.

To elucidate the impact of bta-let-7d on oxidative stress markers, cells were transfected with bta-let-7d mimics and inhibitors, followed by the measurement of SOD, GPx, MDA, and total antioxidant capacity. The results indicated that the bta-let-7d mimics significantly elevated MDA (*p* < 0.05) levels while decreasing SOD (*p* < 0.001) and GPx (*p* < 0.01). Conversely, the inhibition of bta-let-7d resulted in significantly reduced MDA levels (*p* < 0.05) and increased SOD (*p* < 0.0001), GPx (*p* < 0.01), and total antioxidant capacity (*p* < 0.01) (Figure 4A–D). Further quantitative PCR analysis demonstrated that the overexpression of bta-let-7d downregulated Bcl-2 and Caspase-6 while upregulating Bax; the inhibition of bta-let-7d elicited the opposite effects. Additionally, bta-let-7d mimics led to increased expressions of the oxidative-stress-related genes *CAT, NOS2*, and *PTGS2,* while *GPx1* level remained unchanged. In contrast, the bta-let-7d inhibitor resulted in the upregulation of *GPx1* and *NOS2* (Figure 4E). These results indicate that bta-let-7d upregulation exacerbates oxidative stress and apoptotic responses in BEECs.

### 3.5. Interaction Between IGF1R and Bta-Let-7d in the Signaling Pathway

**Figure 5:**
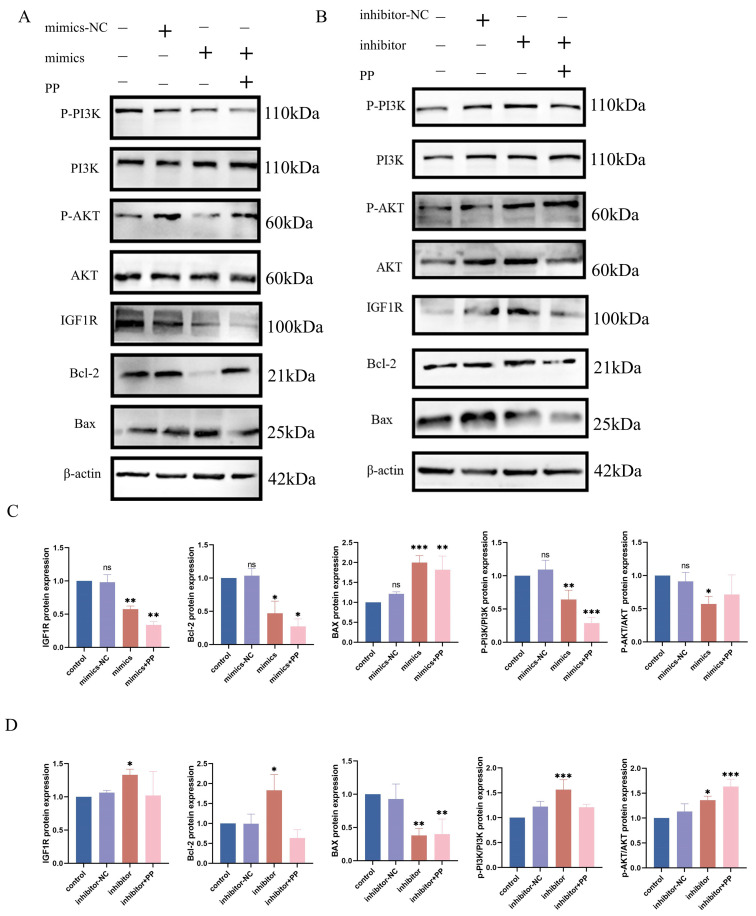
Interaction between IGF1R and let-7d in the signaling pathway. (**A**) BEECs were transfected with bta-let-7d mimics and negative controls, and the expression levels of PI3K pathway-related proteins were detected using Western blotting. (**B**) EECs were transfected with bta-let-7d inhibitor and negative controls, and the expression levels of PI3K pathway-related proteins were detected using Western blotting. (**C**) The grey values of the specified proteins were quantified using ImageJ gel analysis software. (**D**) The grey values of the specified proteins were quantified using ImageJ software. PP stands for potassium permanganate (KMnO_4_). Data are presented as the mean ± SEM of three independent experiments. * *p* < 0.05; ** *p* < 0.01, *** *p* < 0.001.

Western blot analysis showed that bta-let-7d negatively regulates IGF1R expression, thus inhibiting the PI3K/AKT signaling pathway. In cells overexpressing bta-let-7d, the levels of PI3K (*p* < 0.01) and phosphorylated AKT (p-AKT) (*p* < 0.05) were significantly reduced (Figure 5A–C), with downregulated IGF1R expression (*p* < 0.05) positively correlated with PI3K/AKT pathway inhibition. In contrast, IGF1R expression was upregulated upon bta-let-7d inhibition, enhancing PI3K/AKT pathway activation (Figure 5B,D). These results suggest that bta-let-7d suppresses the PI3K/AKT signaling pathway by targeting IGF1R, thereby weakening cell survival and antioxidant capacity.

Further analysis indicates that the inhibition of the PI3K/AKT pathway correlates with an enhanced cellular stress response under oxidative conditions. The upregulation of IGF1R can activate the PI3K/AKT pathway, increasing cellular tolerance to oxidative stress, while the overexpression of bta-let-7d weakens this protective mechanism, rendering cells more susceptible to oxidative damage.

### 3.6. IGF1R as a Target Gene of Bta-Let-7d

**Figure 6:**
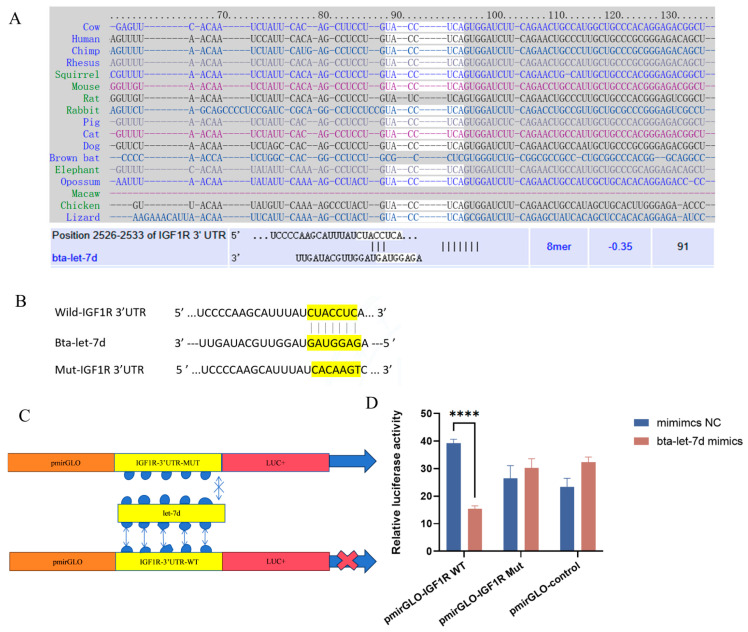
IGF1R is a target gene of let-7d. The text colors in (**A**) and the yellow highlight in (**B**) indicate the binding sites of let-7d with IGF1R. (**A**) Conservation of the let-7d target sequence in IGF1R across different species and the predicted alignment between bta-let-7d and IGF1R 3′-UTR, calculated by TargetScan and miRDB (bottom). (**B**,**C**) Schematic showing the construction of wild-type and mutant plasmids containing the 3′-UTR of IGF1R with or without the predicted bta-let-7d binding site for dual-luciferase reporter assays. (**D**) 293T cells were transfected with bta-let-7d mimics and their negative controls, and luciferase activity was measured using a dual-luciferase reporter assay. Luciferase activity is expressed as the ratio of firefly to Renilla luciferase activity. Concentrations for KMnO_4_ are C1: 0.01%, C2: 0.025%, C3: 0.05%, C4: 0.1%, and C5: 0.5%. Data are presented as the mean ± SEM of three independent experiments. **** *p* < 0.0001.

Bioinformatic analysis identified a list of potential targets of bta-let-7d (Figure 6A). Of note, IGF1R is one of the target genes of bta-let-7d with the most potential (Figure 6A–C). This was further validated by a dual-luciferase reporter assay (Figure 6D): the bta-let-7d mimic significantly inhibited the luciferase activity of IGF1R 3′UTR, with no significant change observed in the mutant vector (Figure 6D). These findings indicate that bta-let-7d directly regulates IGF1R expression by targeting its 3′UTR, playing a role in oxidative stress response.

### 3.7. Negative Regulatory Role of IGF1R in the Oxidative Stress Response

**Figure 7:**
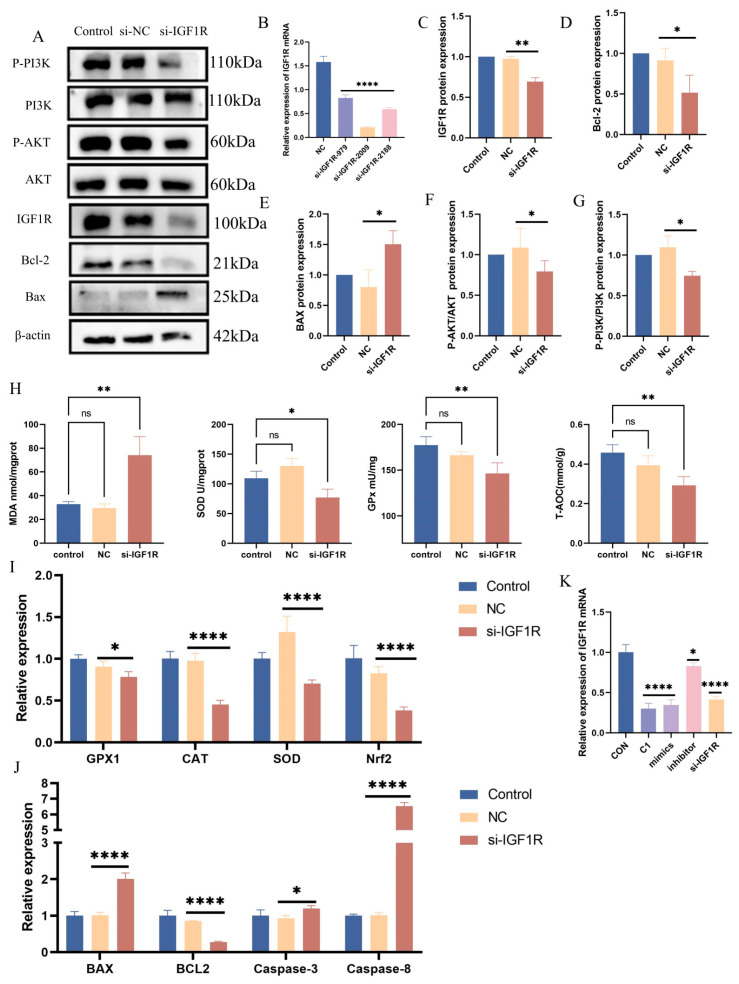
Negative regulation of oxidative stress by IGF1R. (**A**,**C**–**G**) Western blotting was used to detect the protein expression levels of IGF1R, PI3K, phosphorylated PI3K, AKT, and phosphorylated AKT after IGF1R knockdown, with β-actin as an internal control. The grey values of the specified proteins were quantified using ImageJ software. (**B**) RT-qPCR measured IGF1R mRNA levels in BEECs transfected with si-NC or si-IGF1R. (**H**) Total superoxide dismutase (SOD), total antioxidant capacity (T-AOC), lipid peroxidation (MDA), and glutathione peroxidase (GPx) levels were measured in BEECs 24 h after transfection with si-NC or si-IGF1R. (**I**) RT-qPCR was used to measure the relative expression levels of GPx1, CAT, SOD, and Nrf2 in BEECs 24 h after transfection with si-NC or si-IGF1R, with β-actin as an internal control. (**J**) RT-qPCR was used to measure the relative expression levels of Bax, Bcl-2, Caspase-3, and Caspase-8 in BEECs 24 h after transfection with si-NC or si-IGF1R, with β-actin as an internal control. (**K**) RT-qPCR was used to measure the relative expression levels of IGF1R in BEECs treated with 0.01% KMnO_4_ and transfected with either bta-let-7d mimics, bta-let-7d inhibitors, or si-IGF1R. Data are presented as the mean ± SEM of three independent experiments. * *p* < 0.05; ** *p* < 0.01, **** *p* < 0.0001.

To further investigate the role of IGF1R in the oxidative stress response, siRNA knockdown was performed at various IGF1R sites, with si-IGF1R-2009 demonstrating the most significant effect (*p* < 0.0001) (Figure 7B). The knockdown of IGF1R resulted in a marked increase in MDA (*p* < 0.01) levels and a notable reduction in SOD (*p* < 0.05), GPx (*p* < 0.01), and total antioxidant capacity (*p* < 0.01) (Figure 7G). Furthermore, IGF1R knockdown inhibited the PI3K/AKT pathway (*p* < 0.05) (Figure 7A,F,G). Quantitative PCR analyses revealed the downregulation of the antioxidant marker genes GPx1 (*p* < 0.05), CAT (*p* < 0.0001), SOD (*p* < 0.0001), and Nrf2 (*p* < 0.0001), reflecting diminished cellular antioxidant capacity (Figure 7H). Western blot results indicate an upregulation of pro-apoptotic proteins and a downregulation of anti-apoptotic proteins (Figure 7A,D,E). Concurrently, qPCR analysis showed an increased expression of the pro-apoptotic genes Bax (*p* < 0.0001), Caspase-8 (*p* < 0.05), and Caspase-3 (*p* < 0.0001), and a decreased expression of the anti-apoptotic gene Bcl-2 (*p* < 0.0001) (Figure 7I). These findings suggest that IGF1R plays a critical negative regulatory role in the oxidative stress response, where its downregulation exacerbates oxidative stress and apoptotic responses.

### 3.8. Potassium Permanganate-Induced Rat Uterine Damage and IGF1R Expression

**Figure 8:**
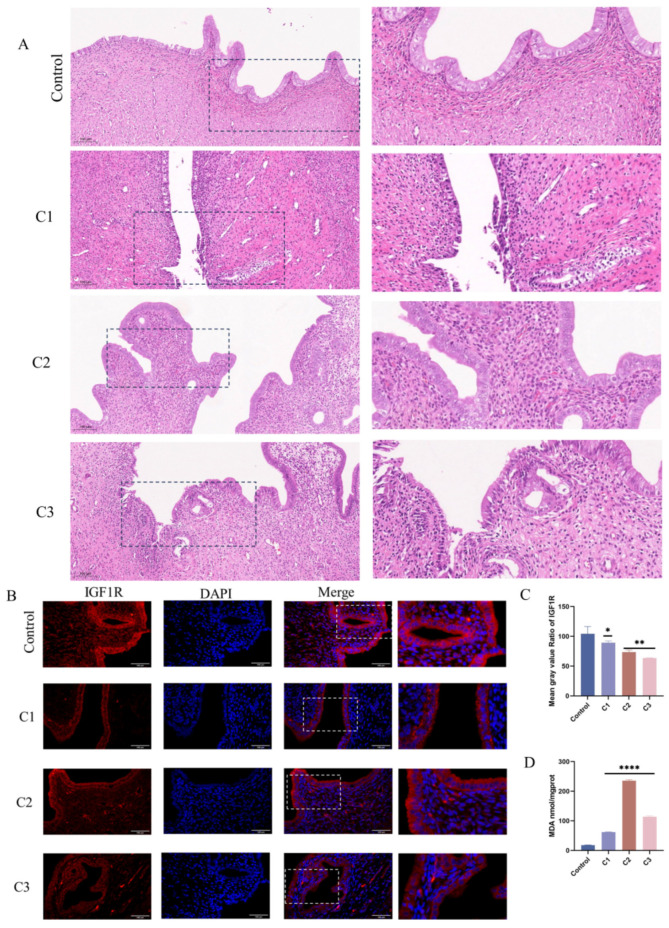
Potassium permanganate-induced rat uterine injury is associated with IGF1R expression. (**A**) Histopathological analysis of rat uterine tissues stained with H&E after potassium permanganate lavage for 12 h (HE, ×100); scale bar = 100 μm. (**B**,**C**) Quantifying IGF1R protein levels and immunofluorescence staining of IGF1R expression in the tissues; scale bar = 100 μm. Blue indicates nuclei and red indicates IGF1R-positive staining. Gray values of the indicated protein were detected using Image J gel analysis software. Data are presented as the mean ± SEM of three independent experiments. (**D**) Lipid peroxidation (MDA) levels in the tissues were measured. Concentrations for KMnO_4_ are C1: 0.01%, C2: 0.025%, and C3: 0.05%. Data are presented as the mean ± SEM of three independent experiments. * *p* < 0.05; ** *p* < 0.01, **** *p* < 0.0001.

Following uterine lavage in rats with varying concentrations of potassium permanganate (C1: 0.01%, C2: 0.025%, C3: 0.05%), histological assessments indicated that the control group’s endometrial structures remained intact, exhibiting no visible damage or inflammation, with normal basal layer arrangement. At concentrations of 0.025% and 0.01% potassium permanganate, slight edema and mild epithelial damage were observed, accompanied by localized inflammatory cell infiltration (Figure 8A). At a concentration of 0.05%, severe tissue damage, edema, and leukocyte infiltration were evident, indicating a pronounced inflammatory response at higher potassium permanganate concentrations. MDA content is elevated to varying degrees in different concentrations of potassium permanganate rinses (*p* < 0.0001), suggesting that elevated potassium permanganate concentrations induce severe oxidative stress (Figure 8D). Immunofluorescence analyses revealed reduced IGF1R expression in endometrial epithelial cells following potassium permanganate exposure compared to the controls (Figure 8B,C). These results indicate that potassium permanganate exposure leads to significant uterine damage and the downregulation of IGF1R expression.

### 3.9. Schematic of IGF1R/PI3K/AKT Pathway Inhibition in Potassium Permanganate-Induced Endometrial Oxidative Stress

**Figure 9:**
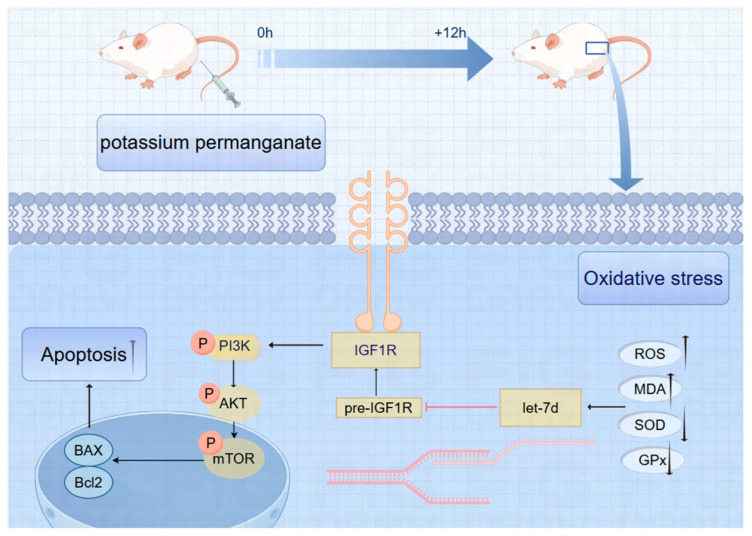
Schematic of IGF1R/PI3K/AKT pathway inhibition in potassium permanganate-induced endometrial oxidative stress.

Potassium permanganate upregulates let-7d and inhibits IGF1R expression, thereby inhibiting the phosphorylation of the PI3K/AKT signaling pathway, ultimately leading to an aggravation of oxidative stress in endometrial cells and the activation of apoptosis programs, as shown in Figure 9.

## 4. Discussion

Potassium permanganate (KMnO4) has long been widely used for wound cleansing and disinfection due to its strong oxidizing properties. KMnO_4_ suppresses bacterial, fungal, and viral growth through its oxidative activity, reducing wound exudation, itching, and inflammatory responses [10]. In veterinary medicine, KMnO_4_ is employed to treat postpartum uterine infections in dairy cows, particularly in cases of endometritis. However, despite its efficacy against bacterial infections, KMnO_4_’s potent oxidizing properties can cause severe damage to healthy tissues. The improper use of KMnO_4_ exacerbates oxidative stress, leading to cell death and tissue injury, especially in sensitive environments like the uterus. Excessive KMnO_4_ induces oxidative stress in endometrial cells, disrupts membrane integrity, and triggers DNA damage and apoptosis [30].

We confirmed that oxidative stress markers, including MDA, SOD, GPx, and T-AOC, are significantly altered in bovine uterine tissues during endometritis, indicating a decline in antioxidant capacity. These findings align with earlier studies showing the role of oxidative stress in tissue damage [5]. Previous studies found that hydroxy-α-sanshool protects hydrogen peroxide-stimulated PC12 cells by modulating the PI3K/Akt pathway to inhibit oxidative-stress-induced apoptosis [18]. We created oxidative stress models using KMnO_4_ in both rat uterine tissues and bovine endometrial epithelial cells (BEECs). The observed oxidative changes align with Li et al.’s hydrogen peroxide-induced model [18], reaffirming KMnO_4_’s ability to induce oxidative stress in BEECs, and our rat model was consistent with Umar et al.’s [30]. Histopathological analysis revealed inflammatory cell infiltration and tissue damage consistent with observations in bovine uteri. Elevated MDA levels confirmed that KMnO_4_ induced oxidative stress and reduced IGF1R expression, detected via immunofluorescence, highlighting its involvement in the oxidative stress response.

MicroRNAs (miRNAs), such as the highly conserved let-7 family, play critical regulatory roles in processes like cell differentiation, cycle regulation, and tissue homeostasis [23]. Specifically, let-7d has been implicated in oxidative stress and apoptosis regulation, with evidence suggesting its ability to modulate antioxidant pathways, as seen in hypothyroid pregnant women [31]. However, its role in bovine endometrial oxidative stress had not been fully explored. Our results show that let-7d is upregulated during KMnO_4_-induced oxidative stress and plays a critical role in regulating antioxidant markers. The transfection of let-7d mimics increased oxidative damage by reducing antioxidant markers (SOD, GPx, T-AOC) and elevating MDA levels, while let-7d inhibitors reversed these effects. This confirms let-7d as a key pro-oxidative regulator.

We further demonstrated that let-7d targets IGF1R, a receptor critical for the PI3K/Akt signaling pathway, which regulates cell survival, apoptosis, and metabolism. In cows with endometritis, let-7d expression was increased while IGF1R expression was decreased, suggesting an inverse relationship. Using dual-luciferase assays, we validated that let-7d directly binds to IGF1R mRNA, suppressing its expression. Transfection experiments confirmed that let-7d mimics downregulate IGF1R mRNA and protein levels, while inhibitors restore IGF1R expression. This indicates that let-7d negatively regulates IGF1R expression, thereby influencing oxidative stress and apoptosis.

In addition to its role in oxidative stress, let-7d promotes apoptosis under KMnO_4_ stimulation. Elevated let-7d expression upregulates pro-apoptotic Bax and downregulates anti-apoptotic Bcl-2, as confirmed by transfection experiments. This pro-apoptotic effect is further linked to IGF1R. IGF1R depletion inhibits the phosphorylation of PI3K and Akt, impairing this pathway’s protective effects and increasing apoptosis markers (e.g., Bax) while reducing anti-apoptotic proteins (e.g., Bcl-2). These findings align with previous studies, such as Olejnik et al.’s [32], who reported similar mechanisms in cardiomyocytes. Together, our results demonstrate that let-7d exacerbates oxidative stress and apoptosis by targeting IGF1R and repressing the PI3K/Akt signaling pathway.

MicroRNAs are known to influence multiple processes in endometrial inflammation and oxidative stress. For example, miR-223 alleviates inflammation by targeting NLRP3 [33], and miR-148a negatively regulates endometritis via TLR4 signaling [34]. In our study, let-7d emerged as a key player in oxidative stress and apoptosis regulation. By targeting IGF1R, let-7d suppresses antioxidant defenses, increases oxidative damage, and promotes apoptosis via the PI3K/Akt pathway. These findings provide novel insights into the molecular mechanisms underlying oxidative stress and apoptosis in bovine endometrial tissues.

## 5. Conclusions

This study highlights the critical role of let-7d in modulating oxidative stress and apoptosis in bovine endometritis. Let-7d exacerbates oxidative damage by targeting IGF1R, suppressing its expression, and impairing PI3K/Akt signaling. These findings suggest that regulating let-7d expression could provide a therapeutic strategy for reducing oxidative stress and improving reproductive outcomes in dairy cows.

## Figures and Tables

**Table 1:** Primers used for qPCR.

Species	Gene Name	Primer Sequence (5′–3′)	GenBank Accession Number	Product Size (bp)
Bos taurus	IGF1R	Forward: CCAAAACCGAAGCTGAGAAG	XM_054377836.1	199
		Reverse: TCCGGGTCTGTGATGTTGTA		
	GPx1	Forward: CTTGCTGCTTGGCGGTCA	NM_174076.3	139
		Reverse: AGGGGAGGCTGGGATGGAT		
	SOD3	Forward: CTTCTTCCACCTTGAGGGCTTC	NM_001428313.1	125
		Reverse: CGGACATCGGGTTGTAGTGC		
	CAT	Forward: CTGGGACCCAACTATCTCCA	NM_001035386.2	148
		Reverse: GATGCTCGGGAGCACTAAAG		
	Nrf2	Forward: GGTTGCCCACATTCCCAAATC	XM_005202314.5	119
		Reverse: CAAGTGACTGAAACGTAGCCG		
	NOS2	Forward: ACCTACCAGCTGACGGGAGAT	XM_024979646.2	316
		Reverse: TGGCAGGGTCCCCTCTGATG		
	Ptgs2	Forward: TCCTGAAACCCACTCCCAACA	NM_174445.2	242
		Reverse: TGGGCAGTCAGGCACAG		
	Bax	Forward: CAGATCATGAAGACAGGGGC	NM_173894.1	389
		Reverse: CGCTCTCGAAGGAAGTCCAA		
	Bcl-2	Forward: ATGTGTGTGGAGAGCGTCAA	NM_001166486.1	143
		Reverse: GGGCCATACAGCTCCACAAA		
	Caspase-3	Forward: AAGCCATGGTGAAGAAGGAA	XM_010820245.4	134
		Reverse: GGCAGGCCTGAATAATGAAA		
	Caspase-9	Forward: CGCCACCATCTTCTCCCTG	XM_024999238.2	83
		Reverse: CCAACGTCTCCTTCTCCTCC		
	β-actin	Forward: CATCACCATCGGCAATGAGC	NM_173979.3	156
		Reverse: AGCACCGTGTTGGCGTAGAG		
	RT-U6	Forward: ACGUGACACGUUCGGAGAATT		
	U6	Forward: CTCGCTTCGGCAGCACA		
		Reverse: AACGCTTCACGAATTTGCGT		

## Data Availability

The datasets generated and analyzed during the current study are available from the corresponding author upon reasonable request. All data materials and software applications support their published claims and comply with field standards.
